# Validation of anthropometric-based weight prediction equations among Ugandan adults: A Cross-sectional study

**DOI:** 10.1371/journal.pone.0306416

**Published:** 2025-12-26

**Authors:** Zakaria Mukasa, Juliet Ntuulo Mutanda, Ronnie Kasirye, Emmanuel Olal, Christopher Lwanga, Victoria Nankabirwa, Fred Nuwaha

**Affiliations:** 1 Makerere University Johns Hopkins University Research Collaboration, Kampala, Uganda; 2 Makerere University College of Health Sciences, School of Public Health, Kampala, Uganda; 3 Uganda Medical Association, Acholi Branch-Gulu, Gulu, Uganda; University Hospital of Padova, ITALY

## Abstract

**Introduction:**

Effective patient management often requires accurate weight estimation. However, the appropriate weight-measuring equipment is not always available in emergencies and low-resource settings. Hence, emergency clinicians resort to less reliable methods of weight estimation, often with negative consequences. In this study, we assess the accuracy of anthropometric-based weight prediction equations in Ugandan adults.

**Methods:**

A cross-sectional study was conducted at Kira Health Center IV. Recruitment was done between 05-01-2022 and 21-02-2022. A sample of 240 adults, 18 years and above, was selected by quota sampling, stratified by sex and nutritional status. Anthropometric measurements, including weight, height, knee height, subscapular skin fold thickness, and circumference measurements, were taken. The predicted weight was computed using the proposed equations, and their accuracy was assessed using Bland-Altman analysis, and the percentage of weight estimates within 10% and 20% of the actual weight.

**Results:**

Out of 240 participants, 50% were females. The median (interquartile range) was 29 (24 –38 ) years for age, 64.5 (54−76) kg, and 162.5 (156.5–170.1) cm, for weight and height, respectively. Rabito equation 3 (Weight = (0.5759x(Mid Arm Circumference)) + (0.5263x(Abdominal Circumference)) + (1.2452x(Calf Circumference)) – (4.8689xSex)-32.9241) was the most accurate, with a percentage of estimates with 10% of the actual weight of 77.08%.

**Conclusion:**

In emergency settings with an absent patient-reported weight or an appropriate weighing scale, using Rabito equation 3 for weight prediction can be used as an accurate alternative. However, fine-tuning will be required before its recommendation for general use.

## Introduction

Measurement of weight is often essential for effective patient management, as safe administration of most medications requires careful tailoring of dosages by clinicians to patients’ weights to minimize overdose-related harm [1,2]. Despite the importance of accurate weight measurement to quality patient care, globally and locally, research shows that many patients seeking care from health facilities are not weighed [3–5]. In 2019, a study in Uganda found that over 98% of patients were not weighed [5]. Moreover, even where scales are available, they may not give accurate weight readings unless they are calibrated [6].

Failure to obtain and use accurate patients’ weights is associated with increased medication dosing errors and subsequently suboptimal treatment, antimicrobial resistance, and drug toxicities [7–9]. A study in Uganda in 2013 found drug overdose to be the most common medication error, accounting for 42.9% of all medication errors [10]. There are several barriers to routine patients’ weight measurement in healthcare practices, including heavy workload, inadequate staffing, inaccurate or a lack of appropriate weighing machines [4,11]. While chair and bedbound scales can be used to obtain the weight of bedridden patients, they are expensive and often not available in resource-limited countries [12].

Over time, several weight prediction techniques like clinicians’ visual weight estimation and the 70 kg standard weight for adults have been proposed [13,14], nevertheless, they often have low accuracy [15]. Patient-reported weight is significantly more accurate than other weight estimation methods [16]; however, it’s not applicable in situations where patients cannot communicate their weight. Anthropometric-based weight prediction, which involves using body size measurements and proportions other than weight to predict weight, is more objective than either clinicians’ visual weight estimation or the 70 kg standard weight for adults and has been employed for a long time. However, the adult anthropometric-based weight prediction equations have mostly been developed in high-income settings [17], and none is well established.

A few pioneering studies have developed and validated weight prediction equations for the sub-Saharan African context [16,18,19]. One successfully validated the use of an anthropometric-based dose for praziquantel among children, improving access in rural populations [18]. However, this tool was used only for praziquantel dosing for children. Kokong tested a weight prediction equation among university students in Nigeria, which showed good accuracy, albeit with sampling limitations [19].

Accurate weight estimation methods are needed in emergency settings and for situations where access to conventional weighing scales is limited. This study aimed to validate anthropometric-based equations for weight estimation among adults in resource-limited settings.

## Materials and methods

### Study setting and design

This was a cross-sectional study conducted at Kira Health Center IV, a high-volume suburban primary healthcare facility in central Uganda, from 05 January to 21 February 2022. The center serves over 100 patients daily, with approximately half of the population being adults aged 18 years and above.

### Participants

All adults (18 years or older), able to stand upright for weight and height measurements, and willing to provide informed consent were included. Those who were pregnant, with altered mentation, and amputated or immobilized were excluded.

### Sample size determination

Using an expected proportion of weight estimates with 10% of the actual weight (P10) of 85%, a margin of error of 5% at a 95% confidence interval, the sample size using Cochran’s formula was approximately 200 participants. Allowing 20% for missing data, a sample size of 240 participants was obtained.

### Sampling procedures

Quota sampling was used, stratified by sex and nutrition status. An equal number of participants were sampled in each stratum using convenience sampling by a research assistant who approached and informed potential participants about the study. Those interested provided written informed consent and had their anthropometric measurements taken. Recruitment for a given stratum was stopped as soon as the target sample size in the stratum was achieved, and overall recruitment stopped when the target sample size was achieved.

### Data sources and measurements

Weight (W) was measured using a flat weighing scale (Seca 761) in kilograms and to the nearest one-tenth of a kilogram (100 grams). Height (H) was measured with a wall-mounted (Seca 206) stadiometer in centimeters(cm) to the nearest millimeter(mm). The Subscapular Skinfold Thickness (SST) was measured using a skinfold caliper (Bozeera skinfold thickness caliper pro) in mm to the nearest mm. The Abdominal Circumference (AC), Calf Circumference (CC), Mid-arm circumference (MAC)/ Mid-Upper Arm Circumference (MUAC), Hip Circumference (HC), and Knee Height (HC) were measured using a flexible non-stretchable measuring tape in cm to the nearest mm.

### Quality assurance and control

A well-trained research assistant with experience in taking anthropometric measurements was used. The tools were tested and calibrated among the patients at Kira Health Center IV before beginning recruitment and set to zero every morning before starting to recruit participants. For uniformity, the SST, MAC, and CC were taken once from the right side for every patient. Data was reviewed for errors by the investigator on the day of data collection.

### Weight prediction methods

10 weight estimation equations from different populations (the Americas, Europe, Africa, and Asia) were selected for validation. Only weight prediction models with variables measured in the study were included in the analysis, and these are summarized in Table 1. The table shows the equations, the population where they were developed, the variables in the equation, and the R^2,^ where available. For the Pediatric Advanced Weight Prediction in the Emergency Room-Extended Length with Mid-Arm Circumference (PAWPER XL-MAC) method modified for adults, the actual tape was not used, but rather, the weight was estimated virtually.

**Table 1:** Weight prediction equations selected for evaluation in the study.

Method	Reference	Population	Variables	Equation	Model
Chumlea (Ch)	[17]	228 elderly (USA)	MAC, CC, SST, and KH	W (Female) = (MAC x 0.98) + (CC x 1.27) + (SST x 0.40) + (KH x 0.87)-62.35	R^2^ = 0.85
				W (Male) = (MAC x 1.73) + (CC x 0.98) + (SST x 0.37) + (KH x 1.16)-81.69	R^2^ = 0.90
Rabito (R)	[2]	368 adults (Brazil)	MAC, AC, CC, SST, and S	W = (0.5030xMAC) + (0.5632xAC) + (1.3180xCC) + (0.0339xSST)-43.1560 **(1)**	R^2^ = 0.93
				W = (0.4808xMAC) + (0.5646xAC) + (1.316xCC) – 42.2450 **(2)**	R^2^ = 0.93
				W = (0.5759xMAC) + (0.5263xAC) + (1.2452xCC) – (4.8689xS)-32.9241 **(3)**	R^2^ = 0.94
Crandall (Cr)	[20]	1471 Obese adults (USA)	MAC and H	W (Females) = −64.6 + (2.15xMAC) + (0.54xH)	R^2^ = 0.55
				W (Males) = −93.2 + (3.29xMAC) + (0.43xH))	R^2^ = 0.59
Lorenz (L)	[21]	6962 adults (German)	AC, H, and HC	W (Males) = −137.432 + (0.60035xH) + (0.785xAC) + (0.392xHC)	R^2^ = 0.85
				W (Females) = −110.924 + (0.4053xH) + (0.325xAC) + (0.836xHC)	R^2^ = 0.82
Kokong (KK)	[19]	122 adults (Nigeria)	H	W = H-100	R^2^ = 1.0
*^a^PAWPER XL-MAC (PXM)	[22,23]	16 years or older (USA)	S, MAC, and H	–	–
Jung (J)	[24]	300 elderly (Hong Kong)	KH, MAC, and Age	W (Males) = KHx0.928 + MACx2.508 – Agex0.144–42.543 W (Female) = KHx0.826 + MACx2.116 – Agex0.133–31.486	R^2^ = 0.81 R^2^ = 0.133
*Cattermole (C)	[25]	8498 children and adults (USA)	MAC	W = (4 x MAC)-50	–

MAC (cm), Mid-arm circumference; AC (cm), Abdominal circumference; CC (cm), calf circumference; SST (mm), Subscapular Skinfold Thickness; S, sex (male = 1 and female = 2); H (cm), height; HC (cm), Hip Circumference; KH (cm), Knee Height; W (kg), Weight. * The model R2 was not specified in the literature. ^a^ Weight estimates were calculated virtually without a tape using an Excel-based model.

### Outcomes

The primary outcome was the proportion of estimates within 10% P10 of the actual weight. A clinically acceptable accuracy for P10 is greater than 70%. The secondary outcomes were the percentage of estimates within 20% of the actual weight (P20), mean percentage error (MPE), and limits of agreement (LOA) obtained from Bland-Altman analysis.

### Data analysis and management

The data were collected on paper case report forms and entered into Microsoft Excel to generate weight estimates using the equations in Table 1. Analysis was done using STATA 17 SE and Medcalc. The continuous variables were summarized using median and interquartile range (IQR) as they were not normally distributed. The LOA were obtained using the Bland-Altman plots with 95% Confidence Intervals (CI). The accuracy of estimates by the various equations was computed using P10 and P20.

### Ethical considerations

Permission was sought from the Makerere University School of Public Health, Higher Degrees Research and Ethics Committee. Administrative clearance was obtained from the District Health Officer (DHO) of Wakiso district and Kira Health Center IV. Written informed consent was sought from the patients before carrying out any study procedures. Only adults were approached for consent; all participants provided consent by themselves, and none required a witness.

## Results

### Description of study participants

A total of 240 participants who fulfilled the eligibility criteria were enrolled in the study. An equal number of males and females were sampled. The variables are summarized in Table 2 below using proportions and Median (IQR).

**Table 2:** Summary of descriptive statistics by Sex.

Continuous Variables			
	All [n (%) or Median (IQR)]	Males [n (%) or Median (IQR)]	Females [n (%) or Median (IQR)]
Sex	240 (100%)	120 (50%)	120 (50%)
Age (years)	29 (24 –38 )	30 (25 –39 )	28 (23 –36 )
Weight (kg)	64.5 (54-76)	67 (56-78)	61.5 (50.5-75.0)
Height (cm)	162.5 (156.5-170.1)	167 (161.1-172.5)	159 (154.9-163.5)
AC (cm)	81.8 (73.3-92.5)	79.5 (73.5-92)	83 (72.5-93.8)
MAC (cm)	28.6 (25-31.6)	28.9 (26.5-31.5)	28 (24.3-32)
KH (cm)	51.5 (48.5-53.2)	52.5 (51.5-54.5)	48.8 (47.5-51.5)
SST (mm)	12 (7 –19 )	10.5 (6 –15)	12 (9 –20 )
CC (cm)	34.5 (31.5-37.5)	35.1 (32.5-38.3)	34.5 (31.5-37.5)
HC (cm)	96.5 (88.8-105.5)	95.4 (88.5-103.3)	100 (89.7-108.8)

AC, Abdominal circumference; MAC, Mid Arm circumference; KH, Knee height; SST, subscapular skinfold thickness; CC, Calf circumference; HC, Hip circumference.

### Primary results

All Rabito equations (1, 2, and 3) had P10 greater than 70 (Table 3); however, none had a P20 greater than 95%. Equation R3 had the least scatter. Overall, Rabito et al. equation 3 (W = (0.5759xMAC) + (0.5263xAC) + (1.2452xCC) – (4.8689xS)-32.9241) was the most accurate based on P10, with Rabito equations 1 and 2 being strong alternatives. These equations also had the highest P20 and the narrowest spread with minimal bias. They had the highest accuracy and precision, low bias, and strong agreement based on the analysis.

**Table 3:** Equations with the outcomes from the different methods of assessment.

Method	P10	P20	MPE	Bland Altman Analysis	
				LLOA	ULOA
R1	76.25	93.75	−1.27	−12.61	15.57
R2	76.67	93.75	−1.38	−12.56	15.78
R3	77.08	94.17	−2.92	−11.15	16.25
Ch	65.00	90.00	−4.06	−12.28	19.08
Cr	30.83	51.25	21.79	−35.85	12.29
L	67.50	88.75	0.10	−15.11	16.56
KK	21.25	43.33	1.51	−33.81	39.29
PXM	52.5	83.75	−0.54	−17.77	21.46
J	45.00	76.25	7.05	−22.89	17.29
C	57.08	85.83	−2.62	−17.51	21.53

LLOA, lower limit of Agreement; ULOA, Upper Limit of Agreement; MPE, Mean Percentage Error; P10 and P20, percentage of estimates within 10% and 20% of the actual weight; R1, Rabtito et al. 1 equation; R2, Rabito et al. 2 equation; R3, Rabito et al. 3 equation; Ch, Chumlea et al. equation; Cr, Crandall et al. equation; L, Lorenz et al. equation; KK, Kokong et al. equation; PXM, PAWPER XL-MAC; J, Jung et al. equation; C, Cattermole et al. equation.

**Fig 1:**
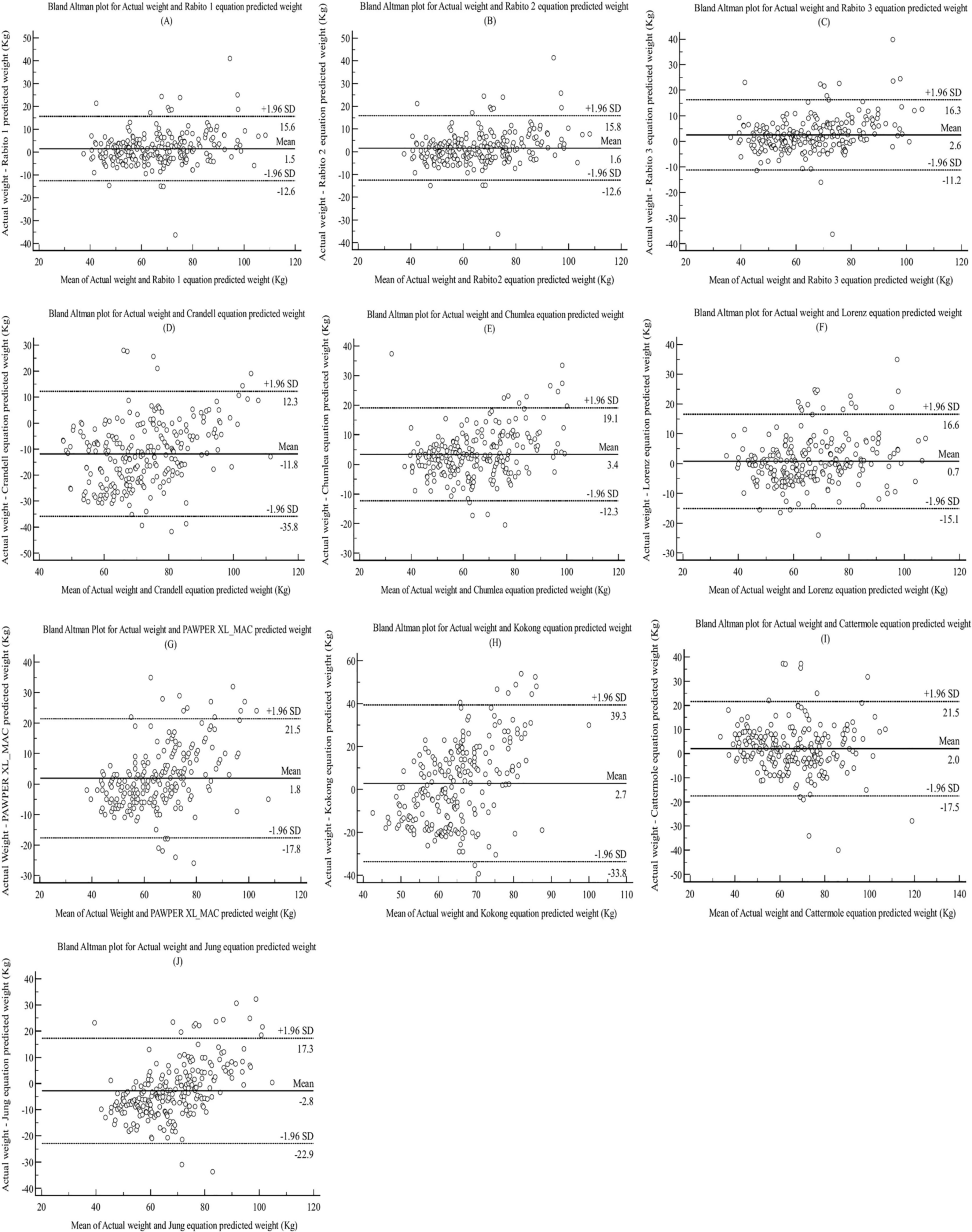
Bland Altman plots comparing the predicted weight by different equations and the Actual weight. The figure shows the Bland-Altman plots for the various equations. Graph A shows the Bland Altman plot between actual weight and weight estimated by Rabito 1 (R1) equation, graph B between actual weight and estimated weight by Rabito 2 (R2), graph C between actual weight and Rabito 3 (R3), graph D between actual weight and weight estimated from Crandall (Cr) equation, graph E between actual weight and weight estimated from Chumlea (Ch) equation and graph F is for actual weight and weight estimated from equations by Lorenz et al **(L)**. Graph G is for actual weight and estimated weight by the PAWPER XL_MAC model. Graph H is for actual weight and estimated weight by the Kokong equation. Graph I is for actual weight and estimated weight using the Cattermole equation. Graph J is for actual weight and estimated weight by the Jung equation. The central dashed line represents the mean difference (bias), and the upper and lower outer parallel dashed lines represent the upper and lower limits of agreement, respectively.

Fig 1 shows Bland Altman plots A to J for actual weight and the various weight prediction equations. The narrower the gap between the LOA (spread), the better the agreement between methods. From the plots, Rabito et al. equations (1, 2, and 3) have a narrow width of agreement and hence better agreement as compared to the other methods.

### Subgroup analysis results

In the subgroup comparison of P10, R2 (W = (0.4808xMAC) + (0.5646xAC) + (1.316xCC) – 42.2450) was the most accurate for females, and R3 for males (Table 4). Among the nutrition categories, the Chumlea et al. equation was the most accurate for the underweight, and the Rabito et al. equation 3 had the most accuracy for the rest of the weight categories. For P20, there was a variation with R3 being the best for underweight and normal categories, and PXM and R1 for overweight and obese, respectively. All equations performed poorly for weight extremes (underweight and obese). All equations systematically overestimated weight at low weight values (underweight) but increasingly underestimated weight at high values of weight (Obese), except the Cattermole equation, which consistently underestimated weight.

**Table 4:** Accuracy of weight prediction equations by Sex and nutritional status.

	R1	R2	R3	Ch	Cr	L	KK	PXM	J	C
Female										
P10	79.17	80.83	74.17	60.0	15.0	71.67	15.0	59.17	50.0	60.83
P20	95.83	95.83	95.58	89.17	27.5	93.33	40.83	85.83	83.33	90.0
Males										
P10	73.33	72.5	80.0	70.0	46.67	63.33	27.5	45.83	40.0	53.33
P20	92.5	91.67	92.5	90.83	75.0	84.17	45.83	81.67	76.25	81.67
Underweight										
P10	73.33	73.33	65.00	76.67	8.33	48.33	0	31.67	10.00	45.00
P20	91.67	91.67	93.33	91.67	23.33	81.67	0	71.67	48.33	83.33
Normal										
P10	81.67	81.67	88.33	70.00	15.00	71.67	56.67	80.00	40.00	61.67
P20	98.33	98.33	100	95.00	36.67	98.33	85.00	98.33	76.67	90.00
Overweight										
P10	76.67	75.00	81.67	65.00	33.33	76.67	28.33	66.67	70.00	56.67
P20	90.00	90.00	90.00	83.33	55.00	91.67	88.33	91.67	95.00	81.67
Obese										
P10	73.33	76.67	77.08	48.33	66.67	73.33	0	31.67	60.00	65.00
P20	95.00	93.33	93.33	85.00	90.00	83.33	0	73.33	85.00	88.33

P10 and P20, percentage of estimates within 10% and 20% of the actual weight; R1, Rabito et al. 1 equation; R2, Rabito et al. 2 equation; R3, Rabito et al. 3 equation; Ch, Chumlea et al. equation; Cr, Crandall et al. equation; L, Lorenz et al. equation; KK, Kokong et al. equation; PXM, PAWPER XL-MAC; J, Jung et al. equation; C, Cattermole et al. equation.

## Discussion

In inpatient care, and more importantly for critically ill, emergency, elderly, long-term bedridden patients, and in community mass drug administration, knowing the accurate body weight of a patient is important. An accurate weight is often essential in critical care to minimize drug overdoses, especially for drugs with a small therapeutic window [26]. Appropriate weighing scales are not always available in emergencies and resource-limited settings to measure patients’ weight. Several studies have evaluated the accuracy and appropriateness of weight estimation equations; nonetheless, only a few have evaluated several equations at once, like in this study, where 10 equations were evaluated at once [27]. The evaluation of several equations enabled a wider options for selection, hence reducing bias. There are two components of accuracy. Trueness, which assesses how close the estimate is to the true value (systematic error), and precision, which assesses the repeatability of the measurements (random error). An overall accuracy of >70% by P10 and >95% by P20 is considered clinically adequate based on previous studies [28,29].

The study evaluated the accuracy of ten weight prediction equations proposed in the literature. The R3 equation was the most accurate based on P10, as well as with P20, and the Bland-Altman analysis. R3 had the highest P10 amongst all equations, closely followed by R2 and R1. The rest of the equations did not reach the clinically significant threshold of P10 > 70%. Although equation R3 uses 4 dimensions (parameters) to estimate weight, it only requires a simple, readily available measuring tape to take the measurements, and these can easily be measured in bedridden patients with minimal turning. The rest of the equations were either significantly less accurate or required specialized equipment, such as the knee height and subscapular skin fold thickness calipers. Height-based equations with 2 or fewer dimensions may be quick to conduct in emergency settings. However, besides the height-based equations being significantly less accurate than R3, the measurement of height may not be feasible in patients with spine deformities.

Among different categories of sex and nutrition status, R3 was the most accurate for all but the two categories of “Females” and “underweight”, where R2 and the Chumlea equation were the most accurate. In 2008, in Brazil, Rabito came to comparable conclusions upon evaluation of anthropometric-based weight prediction equations (R1, R2, and R3) that had been developed earlier in 2006, together with the Chumlea equations [30]. R3 was the best (St. Laurent coefficient 0.8524) out of the three, albeit less accurate than the Chumlea equation. However, the Chumlea equation contains variables that require sophisticated and expensive equipment, such as skinfold thickness calipers and knee height calipers, to measure, limiting its applicability in emergencies and resource-constrained settings.

In Latvia, Balode, and colleagues found the R1 equation to be the most accurate [27], different from the results obtained from this study, however, there were differences in the measure of accuracy (mean difference) used and the population distribution in the study sample. Their population was made predominantly of elderly females, versus young adults of both sexes in equal numbers in our study. Several studies have shown that there are gender [31,32] and age [33] differences in anthropometric dimensions.

Kokong et al.‘s equation was developed in Nigeria, in a population with similarities to the Ugandan one. The Kokong equation, together with the Crandall equation, performed the worst among all the assessed methods, similar to what was found by Cattermole et al. when they assessed several weight prediction equations using data from the National Health and Nutrition Examination Survey [28]. The two equations performed the worst with P10 of 34.5% and 26.4% and P20 of 63.4% and 52.1% for Crandall and Kokong, respectively. The Kokong equation performed very poorly at the extremes of nutritional status. The equation is entirely based on height, which remains constant in adults regardless of the weight changes, which explains its poor performance. More recently, the PAWPER XL-MAC method was evaluated among Rwandese adolescents, and 91.3% of the weight estimates fell within 20% of the actual weight, and 83.7% in adults [16]. This is comparable to what we found in this study (83.75%) but less than the 88.4% found by Cattermole et al.[28]. However, all estimates were less than the clinically acceptable 95%.

Generally, most of the equations overestimated the weight. This was different from what was found in Nigeria by Kokong and colleagues, where their novel weight prediction method overestimated the weight [19]. All equations performed poorly in extremes of nutritional status (obese and underweight) individuals compared to those in other nutrition categories. The Crandall equation performed relatively well in the obese category compared to other categories, which is not surprising since the equation was developed among obese adults [20].

### Strengths and limitations

A major strength of this study was the stratification of study participants by nutritional status and sex. Stratification by nutritional status made it possible to obtain enough participants in the extremes of nutritional statuses that would otherwise be underrepresented with non-stratified sampling. This enabled us to assess how the proposed equations performed in these extremes. Stratifying by sex also enabled us to assess how the performance of the equations was affected by sex.

The study also had several limitations. First, there was potential selection bias due to the use of non-probability sampling techniques. The sample ultimately had predominantly young adults, hence not representative of all adults, especially the elderly bedridden patients on whom the equations may be applied. Secondly, there was a possibility of measurement error and measurement bias, since one person performed the measurement, and measurements were performed once. However, this was reduced by using trained health workers and using calibrated and standard equipment. All the measurements were taken in an upright position, yet for bedridden patients, we use measurements taken in the supine position. It was not possible to make comparisons with some methods of weight prediction, such as the Mercy tape method, clinician and patient estimated weight, as the data required to compute these methods was not collected. The weight estimates for the PAWPER XL MAC methods were computed using an Excel-based formula rather than the actual tape, which is different from real-life emergency settings. In addition, Bland-Altman analysis was used despite the data not fulfilling the assumption of normality of the difference between methods.

## Conclusions and recommendations

Equation R3 was the most accurate overall, based on the primary outcome. In situations where patient-reported weight is not available and patients’ weight cannot be measured, equation R3 is recommended for use to predict the weight of young adult patients with relative confidence. Equation R3 only requires a measuring tape, which is cheap and readily available. Equations R1 and Ch require skin fold thickness and knee height calipers to compute and, therefore, would not be very easily applicable in resource-limited settings.

Further future research should aim at evaluating these equations in the elderly population and or develop equations tailored for the Ugandan and sub-Saharan African population. This equation could also be digitalized and incorporated into a phone-based application that can be used to predict weight.

## Supporting information

S1 File Checklist. STROBE checklist v4 cross-sectional.
(PDF)

S2 Data Study Data. (XLSX)
